# Diagnostic accuracy of multiparametric magnetic resonance imaging in detecting extracapsular extension in intermediate and high - risk prostate cancer

**DOI:** 10.1590/S1677-5538.IBJU.2016.0485

**Published:** 2018

**Authors:** Cristina Dominguez, Mauricio Plata, Juan Guillermo Cataño, Mauricio Palau, Diego Aguirre, Jorge Narvaez, Stephanie Trujillo, Felipe Gómez, Carlos Gustavo Trujillo, Juan Ignacio Caicedo, Camilo Medina

**Affiliations:** 1 Department of Urology, Hospital Universitario Fundación Santa Fe de Bogotá, Colombia, CO; 2 Department of Pathology, Hospital Universitario Fundación Santa Fe de Bogotá, Colombia, CO; 3 Department of Radiology, Hospital Universitario Fundación Santa Fe de Bogotá, Colombia, CO

**Keywords:** Prostatic Neoplasms, Magnetic Resonance Imaging, Prostatectomy

## Abstract

**Objectives:**

To evaluate the diagnostic performance of preoperative multiparametric magnetic resonance imaging (mp-MRI) as a predictor of extracapsular extension (ECE) and unfavorable Gleason score (GS) in patients with intermediate and high-risk prostate cancer (PCa).

**Materials and Methods:**

Patients with clinically localized PCa who underwent radical prostatectomy (RP) and had preoperative mp-MRI between May-2011 and December-2013. Mp-MRI was evaluated according to the European Society of Urogenital Radiology MRI prostate guidelines by two different readers. Histopathological RP results were the standard reference.

**Results:**

79 patients were included; mean age was 61 and median preoperative prostate-specific antigen (PSA) 7.0. On MRI, 28% patients had ECE evidenced in the mp-MRI, 5% seminal vesicle invasion (SVI) and 4% lymph node involvement (LNI). At RP, 39.2% had ECE, 26.6% SVI and 12.8% LNI. Sensitivity, specificity, accuracy, positive predictive value (PPV), and negative predictive value (NPV) of mp-MRI for ECE were 54.9%, 90.9%, 76%, 81% and 74.1% respectively; for SVI values were 19.1%, 100%, 77.3%, 100% and 76.1% respectively and for LNI 20%, 98.4%, 86.7%, 66.7% and 88.7%.

**Conclusions:**

Major surgical decisions are made with digital rectal exam (DRE) and ultrasound studies before the use of Mp-MRI. This imaging study contributes to rule out gross extraprostatic extension (ECE, SVI, LNI) without competing with pathological studies. The specificity and NPV are reasonable to decide surgical approach. A highly experienced radiology team is needed to provide accurate estimations of tumor extension and aggressiveness.

## INTRODUCTION

Risk stratification for localized PCa is a combination of multiple clinical and laboratory parameters, none of which includes an imaging test providing adequate anatomical detail. These parameters are used to classify patients into risk groups along with nomograms that predict outcomes such as pathological staging, biochemical recurrence, clinical progression and cancer specific survival. More than a third of patients are misclassified with clinical staging, PSA and transrectal ultrasound-guided (TRUS) prostate biopsy (1). Recently, additional parameters such as MRI and the percentage of positive biopsy cores have been added into risk classification methods, both with promising results (2).

Traditionally, RP and radiotherapy have been considered the reference standard treatment for patients with localized PCa (3) both achieving long-term disease control. High rates of erectile dysfunction and incontinence are related to surgical techniques and difficult preservation of the neurovascular bundles. Therefore, accurate preoperative knowledge of tumor stage and possible ECE is crucial in achieving the best surgical, oncological and functional results (4).

Recent findings support the use of mp-MRI of the prostate, combining T2-weighted imaging with diffusion-weighted imaging (DWI) and perfusion imaging, as the most sensitive and specific imaging tool for different clinical scenarios in patients with PCa such as detection, staging, and follow-up (5-13). Nonetheless, its routine use is still a topic of debate given the high variability among studies regarding the diagnostic accuracy of mp-MRI in staging and prediction of ECE (14).

The aim of this study was to evaluate diagnostic accuracy of a 1.5 tesla mp-MRI in detecting ECE, SVI, LNI and unfavorable GS in patients with intermediate and high-risk PCa.

## MATERIALS AND METHODS

After approval from our hospital review board and ethics committee, clinical records were reviewed retrospectively. Patients with clinically localized PCa, who underwent RP and extended lymph node dissection between May 1st 2011 and December 31st 2013 at our institution, and had preoperative mp-MRI, were identified. Inclusion criteria comprised intermediate or high-risk cancer patients as defined by D'Amico classification (intermediate risk: clinical stage T2b or PSA levels between 10.1 and 20ng/mL or GS 7; high risk: clinical stage ≥T2c or PSA levels >20ng/mL or GS 8-10). Finally, the diagnostic accuracy of mp-MRI in detecting ECE, SVI, LNI and unfavorable GS (equal or greater than 8) was analyzed.

### Multiparametric MRI and Image analysis

**Figure 1:**
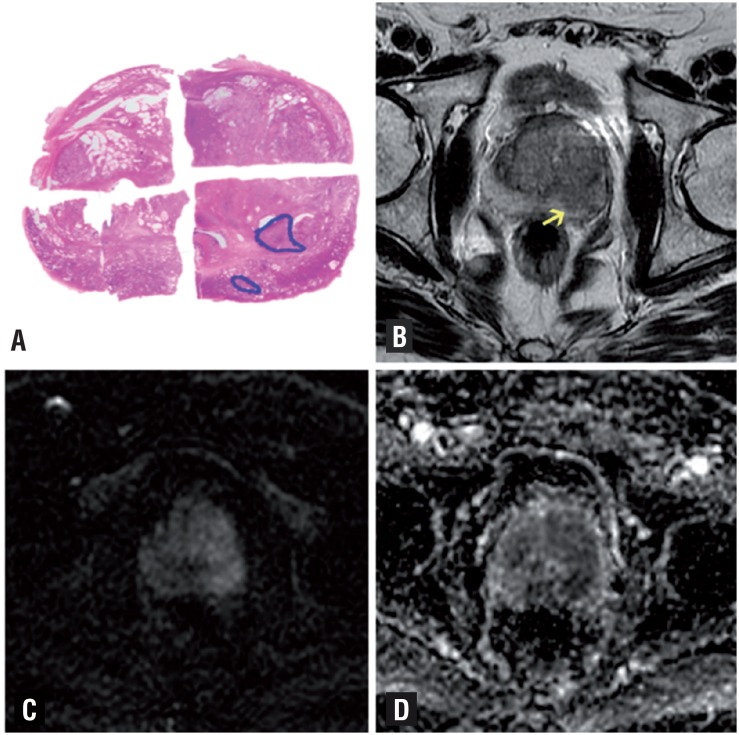
Male, 69-year-old patient with prostate cancer Gleason 8 (4+4). (a) RP specimen shows index lesion of 22mm, Gleason 4+5=9 prostate cancer. MRI demonstrate low signal 12mms lesion on T2w images (arrow) in the peripheral zone confined to the prostate (b), there is a focal area of hyper signal in dwi with b value =800 (c) and reduced ADC (d). No extracapsular extension is seen in MRI. PIRADS: T2WI=4/5; DWI=4/5.

**Figure 2:**
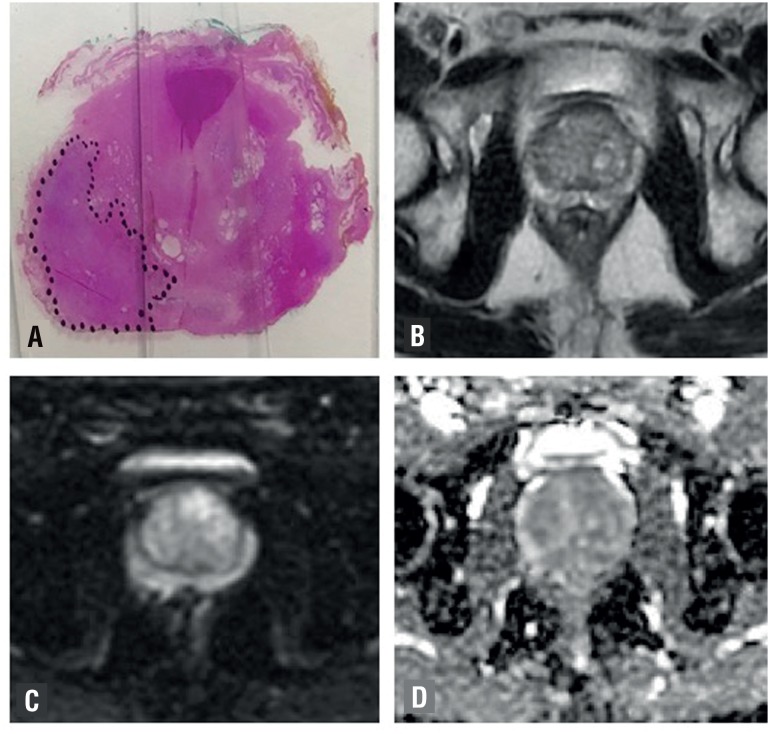
Male, 61-year-old patient with prostate cancer Gleason 7 (4+3) with ece. (a) RP specimen shows index lesion of 30 mm on left base of the prostate. MRI demonstrates heterogeneous and high signal multinodular in T2w sequences (b), there is no hypervascular lesions and no focal areas of restriction on DWI (c) and ADC images (d). No extracapsular extension is seen on MRI. PIRADS score on MRI: T2WI = 2/5; DWI = 2/5.

Preoperative mp-MRI was performed with a 1.5 tesla Siemens system. Patient should wait 6 weeks after biopsy for MRI study; no rectal preparation nor endorectal coil were used in any patient. Studies included T2-weighted imaging, dynamic contrast-enhanced imaging and diffusion-weighted imaging. Mp-MRI results were assessed and reported according to the European Society of Urogenital Radiology (ESUR) MRI prostate guidelines from 2012 by two different radiologists with different years of experience (14 and 8 years of experience). Readers knew diagnosis and initial PSA level but were blinded to details of histopathological report. They adopted one of the following image signs to subjectively classify ECE: 1) bulging of prostatic contour; 2) irregularity of prostatic contour; 3) neurovascular bundle thickening; 4) loss or discontinuity of prostatic contour line; 5) measurable extra-capsular disease. The sum of three of the previously mentioned signs were considered positive for ECE. Tumor contact length (TCL) to the prostate contour was also measured and a 12mm-threshold was used as an additional sign of ECE and independently analyzed as a different variable. For SVI, the subjective image signs adopted were: 1) expansion; 2) low T2 signal; 3) filling in of angle; 4) enhancement and restricted diffusion. The sum of two signs were considered positive for SVI. For LNI, size criteria were used and a short axis >10mm was considered positive (15). For case examples, see Figure-1 and Figure-2. Apparent diffusion coefficient (ADC) is the measure of the magnitude of diffusion of water molecules within tissue. These values were obtained by positioning the region of interest (ROI) in the index lesion (defined as the largest lesion) and a threshold of less than 0.87×10-3mm2/s was considered indicative of unfavorable GS.

### Histopathological evaluation

RP specimens were macroscopically marked with ink and fixed in formalin. The specimen was serially sectioned at 3mm thickness. Perpendicular cuts were done for the first portion of the apex and base, the remaining prostate was then cut in the sagittal plane and, posteriorly, in quadrants from apex to base. Slices were further cut into microscopic sections of 3-5μm and stained with Haematoxylin and Eosin. ECE was defined as tumor cell growth into the extraprostatic tissue and subclassified as: 1) focal ECE when involving only a few glands or a tumor involving less than one high power (40X) field in one or two sections or 2) stablished ECE when a more extensive spread was seen beyond the prostatic edge (16). SVI was defined as tumor infiltration of the muscular wall of the seminal vesicles. Lymph nodes were processed as a whole with posterior differentiation of nodules from fat. The pathological T-stage (pT) was defined according to the TNM classification.

### Statistical analysis

Socio-demographic characteristics were described. A descriptive analysis was conducted and the measures of central trend and variability were reported. Median or mean for continuous variables and absolute or relative frequencies for categorical variables were calculated. Contingency tables (using most experienced radiologist data) were used to calculate overall diagnostic accuracy, sensitivity, specificity, positive predictive value and negative predictive value of mp-MRI in predicting ECE, SVI, LNI and GS greater or equal to 8. Finally, a receiver operating characteristic curve (ROC curve) with an area under the curve (AUC) values were generated to analyze predictive accuracy of ADC in detecting unfavorable GS, and Spearman correlation was used. Inter-reader reliability was calculated using kappa statistics (0-0.2 none, 0.21-0.39 minimal, 0.4-0.59 weak, 0.6-0.79 moderate, 0.8-0.9 strong, above 0.9 almost perfect). A p-value below 0.05 was considered significant. The analysis was performed using STATA 13.1 software.

## RESULTS

Seventy-nine patients were eligible for the study; patient and disease characteristics are shown in Table-1. 36.7% of the patients had a PSA >10ng/mL. Mp-MRI quality was adequate for interpretation; seven subjects had more than 50% hemorrhage image changes from prior biopsy without significant implications for reading. 21 (28%) patients had ECE evidenced by the mp-MRI, 4 (5.3%) patients had SVI and 3 (4%) patients had LNI. RP specimen analysis reported 31 (39.2%) patients with ECE, 21 (37.5%) patients with SVI and 10 (12.9%) with LNI. Measurement of diagnostic agreement between both radiologists for ECE, SVI and LNI reported kappa values of 0.287, 0.5726 and 0.380 respectively, demonstrating minimal and weak agreement when two radiologists with different years of experience assessed all three variables.

**Table 1:** Patient and disease characteristics.

Characteristic		Value
Patient Age (years)*		61.1 (39-78, SD±7.50)
PSA Level (ng/mL)^		7.0 (0.02-31, SD±7.25)
**Clinical Stage** n		
	T1c	57 (74.0%)
	T2a	17 (22.1%)
	T2b	2 (2.6%)
	T3a	1 (1.3%)
**D’Amico classification** n		
	Intermediate risk	51 (65.4%)
	High risk	27 (34.6%)
**pathological stage** n		
	pT2	46 (58.2%)
	pT3a	13 (16.5%)
	pT3b	20 (25.3%)
**Biopsy Gleason Score** n		
	6	6 (7.7%)
	7	46 (59%)
	8	20 (25.6%)
	9	5 (6.4%)
	10	1 (1.3%)
**pathology Gleason Score** n		
	6	2 (2.5%)
	7	53 (67.1%)
	8	14 (17.7%)
	9	10 (12.7%)

* mean and (range, standard deviation);

^ median and (range, standard deviation);

n patient number

**Figure 3:**
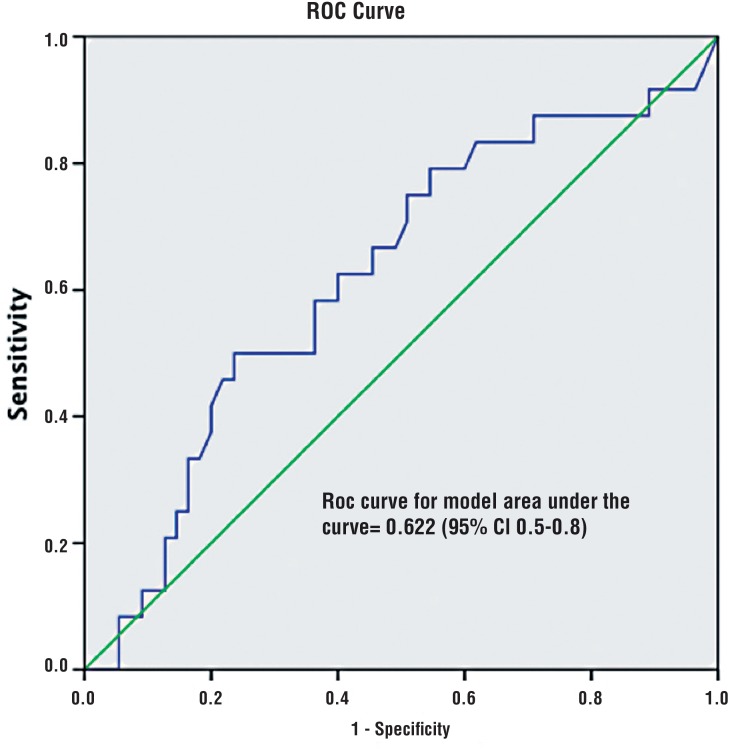
ROC of ADC numeric values in predicting unfavorable GS. AUC value is shown.

Mp-MRI findings were compared to final pathology. Results of accuracy, sensitivity, specificity, PPV and NPV of mp-MRI in detecting ECE, SVI and LNI in comparison to histopathological results are summarized in Table-2. AUC for ECE was 0.7 (95% CI 0.6-0.8), for SVI 0.6 (95% CI 0.5-0.7) and for LNI 0.6 (CI 0.5-0.7) (Figure-3).

**Table 2:** Comparison of operative characteristics of mp-MRI in high risk PCa for ECE, SVI, LNI compared to pathology results.

Study		Sample size (n)	Sensitivity (%)	Specificity (%)	Accuracy (%)	PPV (%)	NPV (%)
**Jeong et al.** (13)^		922					
	ECE		43	84	61	79	52
	SVI		35	94	81	62	83
	LNI		14	97	92	23	95
**Lista et al.** (24)*		85					
	ECE		58	98	-	95	75
	SVI		75	96	-	80	94
**Pinaquy et al.** (25)*		47					
	ECE		72	77	-	86	59
	SVI		73	95	-	95	73
	LNI		33	91	-	50	84
**Cerantola et al.** (26)^T^		60					
	ECE		35	90	62	79	57
**Boesen et al.** (4)^T^		87					
	ECE		74	88	83	77	86
**Somford et al.** (16)^T^		183					
	ECE		64.9	72.7	-	88.9	38.1

* 1.5T MRI scanner;

T 3.0T MRI scanner;

^ Overall using both 1.5T and 3.0T MRI scanner

When focal and established ECE were considered separately, accuracy, sensitivity, specificity, PPV and NPV were 73%, 53.9% (95% CI 42.6-65.1), 77.4% (95% CI 68-86.9%), 33.3% (95% CI 22.7-44), 88.9% (95% CI 81.8-96) for focal ECE, and 73%, 52.6% (95% CI 41.3-63.9), 80.4% (95% CI 71.3-89.4), 47.6% (95% CI 36.3-58.9), 83.3% (95% CI 74.9-91.8) for established ECE.

With mp-MRI tumor contact length (TCL) threshold of 12mm for the diagnosis of ECE, we found accuracy, sensitivity, specificity, PPV and NPV values of 52.7%, 69.2% (95% CI 58.7-79.8), 49.2% (95% CI 37.8-60.6), 22.5% (95% CI 13-32) and 88.2% (95% CI 80.9-95.6) for focal ECE, and of 55.4 %, 68.4% (95% CI 57.8-79), 50.9% (95% CI 39.5-43.1), 32.5% (95% CI 21.8-43.2) and 82.4% (95% CI 73.7-91) for established ECE, respectively.

Finally, when ADC values were compared to pathological results, mp-MRI accuracy, sensitivity, specificity, PPV and NPV in predicting unfavorable GS were 69.3%, 13% (95% CI 5.4-20.7), 94.2% (95% CI 88.9-99.5), 50% (95% CI 38.7-61.3) and 71% (95% CI 60.8-81.3), respectively. When ADC values were considered as a continuous variable, AUC was 0.622 (95% CI 0.5-0.8) (Figure-1) and the Spearman's Rho was −0.34 (p0.001).

## DISCUSSION

Identification of occult ECE, SVI and LNI is of critical importance for prognosis and treatment selection of patients with intermediate and high-risk disease PCa. Clinical staging in this study was predominantly T1c and T2a (74% and 22.1%) the remaining being classified as T2b and T3a. Pathological staging reported pT2 in 58.2% of the cases, pT3a in 16.5% and pT3b in 25.3%. Pathological ECE was reported on 39.2% of the cases and 30.4% had an unfavorable GS (≥8). Understaging is frequent with only clinical assessment (15), therefore, it underscores the importance of a correct staging and identification of intermediate and high-risk patient preoperatively, which may lead to improved decision-making regarding treatment and surgical approach.

Operative characteristics of mp-MRI for the detection of ECE, SVI and LNI, differ among published studies and are highly influenced by the use of endorectal coil (er), MRI parameters and clinical interpretation (16). Recent reports are focused on improving er-MRI imaging technology and functional imaging techniques (dynamic contrast enhancement and diffusion weighted imaging) to optimize accuracy in staging suspicious lesions (17). These advances lead to the use of combined mp-MRI with anatomic T2-weighted imaging, with promising results for PCa detection and extension. One meta-analysis (18) determined diagnostic accuracy of mp-MRI for PCa detection using anatomic T2-weighted imaging combined with two functional techniques (DWI and DCE-MRI). Pooled data of seven studies (526 patients) showed sensitivity of 0.74 (95% CI, 0.66-0.81), specificity of 0.88 (95% CI, 0.82-0.92) for PCa detection, with NPVs ranging from 0.65 to 0.94.

Our results are consistent with those reported in the literature. For ECE detection, mp-MRI has a reported sensitivity between 35-62.5% and specificity of 89-92% (Table-2). Our study showed sensitivity of 55% and specificity of 91%, both within the range of the literature. Feng et al. (15) sought to evaluate mp-MRI ability to detect focal or established ECE reported by pathology. When considered separately, mp-MRI had a low performance in identifying focal ECE, with a sensitivity of 14.3% and a PPV of 5.6%. However, mp-MRI was accurate in predicting established ECE, with a sensitivity of 73% and a PPV of 57%. Our study reported a sensitivity of 53% and a PPV of 48% for established ECE, and for focal ECE performance was poor. Mp-MRI is unable to identify and localize focal ECE, which accounts for a large majority of the cases (18). Nerve-sparing RP requires of mp-MRI to provide a high NPV in detecting ECE. Our results for SVI and LNI are also comparable to literature (Table-2). SVI detection has been reported with PPVs of 62% to 95% and NPVs of 73% to 83%. Sensitivity and specificity for LNI is between 14-33% and 91-97%, respectively (19).

Measurement of diagnostic agreement between radiologists in our study for ECE, SVI and LNI demonstrated minimal and weak. It has been demonstrated in literature that inter-reader reproducibility tend to be higher for relatively experienced readers. This can be explained due to study complexity, different reading scales used such as the PI-RADS of Likert scale and interpretation sensibility may be affected by lesion location, volume and tumor aggressiveness (20, 21).

Furthermore, TCL parameter has also shown correlation with pathologically confirmed microscopic ECE. Baco et al. (22) documented accuracy of 82%, 79% sensitivity, 85% specificity, 76% PPV and 88% NPV when using a 20mm TCL threshold. Our study evaluated the differences in predictive values by using a 12mm threshold when comparing established and focal microscopic ECE, both with acceptable NPVs when evaluating these parameters. Other studies have concluded that a higher length of contact between tumor margin and prostatic capsule is associated with an increased risk of ECE.

In general, literature concludes that mp-MRI has a fair performance capacity for predicting unfavorable GS. Hegde et al. (12) conducted a retrospective study with 118 patients, demonstrating that ECE or SVI findings on mp-MRI are associated with a higher risk of identifying previously undetected GS 8-10 disease. In our study, mp-MRI evidenced low power either to confirm or rule out unfavorable GS. Other studies have evidenced that a high percentage of patients have GS ≥7 at non-index tumors and mp-MRI has low power for the detection of these lesions (23). Also, the diffusion coefficient ADC allows for quantitative and qualitative assessment of tumor aggressiveness and has positive correlation with GS (24). In this study, ADC as binary variable when compared to GS >=8 had high specificity and NPV, low AUC and its values are inversely correspondent to GS as reported with Spearman rho, although it shows a low negative correlation. Mp-MRI has shown to be useful in identifying PCa extension and especially in ruling in locoregional extension.

The limitation of this study is its retrospective nature and small sample size leading to large confidence intervals. Also, operative characteristics depend on how the presence of ECE, SVI and LNI are defined. For our study, the presence of locally advanced disease consisted of binary variables (yes or no), and the data would be improved by using ordinal scales such as prostate imaging reporting and a standardized data system (PIRADS) for extension evaluation. According to the magnetic field strength, the 3T magnet increases signal to noise ratio therefore increases resolution. However, both 1.5T and 3.0T can provide accurate and reliable diagnostic exam when technical parameters are properly used (23). Although members of the PIRADS steering committee prefer the use of 3T, 1.5T magnets are more widely available and our study reflects the practice of most of the devices currently installed worldwide. Finally, both radiologists had a 10-year experience difference which makes data heterogeneous and consequently the low interrater reliability.

## CONCLUSIONS

Major surgical decisions are made with DRE and ultrasound studies before the use of Mp-MRI. This imaging study contributes to rule out gross extraprostatic extension (ECE, SVI, LNI) without competing with pathological studies. The specificity and NPV are reasonable to decide surgical approach. A highly experienced radiology team is needed to provide accurate estimations of tumor extension and aggressiveness. Moreover, it is necessary to carry out prospective and multicenter studies in order to achieve higher consensus regarding MRI use in PCa assessment in developing countries.

## ABBREVIATIONS

RP: = Radical prostatectomy
PSA: = Prostate specific antigen
Pca: = Prostate cancer
GS: = Gleason score
TRUS: = Transrectal ultrasound-guided
MRI: = Magnetic resonance imaging
TCL: = Tumor contact length
AUC: = Area under the curve
cT: = Clinical tumor stage
Mp-MRI: = Multiparametric MRI
PIRADS: = Prostate imaging reporting and data system
T2W: = T2-weighted
DWI: = Diffusion-weighted imaging
DCE-MRI: = Dynamic contrast-enhanced MRI
ADC: = Apparent diffusion coefficient
DCE: = Dynamic contrast-enhanced
ECE: = Extracapsular extension
SVI: = Seminal vesicle invasion
LNI: = Lymph node involvement
